# Supplementary material for: Analytical Validation of a Direct Competitive ELISA for Multiple Mycotoxin Detection in Human Serum
Source: Toxins (Basel). 2022 Oct 25;14(11):727. doi: 10.3390/toxins14110727 (PMC9694295; doi:10.3390/toxins14110727)
Supplement: Supplementary file 1 [file toxins-14-00727-s001.zip › toxins-1974591-supplementary.pdf]

Supplementary Material: Analytical Validation of a Direct Competitive ELISA for Multiple Mycotoxin Detection in Human Serum

Kunal Garg, Fausto Villavicencio-Aguilar, Flora Solano-Rivera and Leona Gilbert

Table S1. The serum from a healthy donor does not interfere with the recovery and matrix analysis for Aflatoxin B1 (AFB1), deoxynivalenol (DON), fumonisin (FUM), ochratoxin A (OTA), and zearalenone (ZEA). Non-spiked serum pre-treated and extracted with 1% formic acid in acetonitrile provides similar optical density (OD) values to non-spiked 2% BSA. The variation (%CV) between non-spiked serum and 2% BSA is less than 10% and suggests that serum from the healthy donor is negative for AFB1, DON, FUM, OTA, and ZEA.

**Mycotoxin-HRP**	**Non-spiked serum**		**Non-spiked 2% BSA**		**%CV**
	**Repeat 1**	**Repeat 2**	**Repeat 1**	**Repeat 2**	
**AFB1-HRP**	4.000	4.000	4.000	4.000	0.000
DON-HRP	3.033	2.496	2.635	2.833	8.523
FUM-HRP	2.298	2.129	2.239	2.382	4.700
OTA-HRP	4.000	3.534	3.650	3.645	5.459
ZEA-HRP	3.454	2.891	3.468	3.659	9.842

Table S2. The assay performance from the present study is being compared to commercial tests and emerging methodologies optimized in various matrices for Aflatoxin B1 (AFB1), deoxynivalenol (DON), fumonisin (FUM), ochratoxin A (OTA), and zearalenone (ZEA). The assay performances compared in tables 2 and 3 should not be considered a head-to-head comparison but a comparison between detection techniques that have employed similar formats for establishing detection (i.e., direct detection) and calibration curves (i.e., non-linear).

	**AFB1**	**DON**	**FUM**	**OTA**	**ZEA**
Present study	Human serum	Human serum	Human serum	Human serum	Human serum
Cusabio	Edible oil	Feedstuff and cereals	Feedstuff and corn	Wheat, corn, etc	Wheat, corn, etc
Elabscience	Wheat	Feedstuff and cereals	Edible oil	Cereals	Cereals
Helica™	Corn	Wheat and corn	Barley, corn, etc	Milk, serum, etc	Barley, rice, etc
AgraQuant®	Feedstuff, cereals, etc	Feedstuff, cereals, etc	Feedstuff, cereals, etc	Feedstuff, cereals, etc	Feedstuff, cereals, etc
VICAM	Feedstuff	Cereals	Corn	Wheat and coffee	Barley, rice, etc
Wu et al. 2022 [33]	Corn	Corn	Corn	Corn	Corn
Xing et al. 2020 [34]	Wheat	Wheat	Wheat	Wheat	Wheat
Charlermroj et al. 2021 [35]	Buffer	Buffer	Buffer	Buffer	Buffer
Joshi et al. 2016 [36]	Barley	Barley	Barley	Barley	Barley
Wie et al. 2019 [37]	Wheat and corn	Wheat and corn	Wheat and corn	Wheat and corn	Wheat and corn

Reference

33. Wu, Y.; Zhou, Y.; Huang, H.; Chen, X.; Leng, Y.; Lai, W.; Huang, X.; Xiong, Y. Engineered Gold Nanoparticles as Multicolor Labels for Simultaneous Multi-Mycotoxin Detection on the Immunochromatographic Test Strip Nanosensor. *Sens. Actuators B Chem*. **2020**, *316*, 128107. https://doi.org/10.1016/j.snb.2020.128107.

34. Xing, C.; Dong, X.; Xu, T.; Yuan, J.; Yan, W.; Sui, X.; Zhao, X. Analysis of Multiple Mycotoxins-Contaminated Wheat by a Smart Analysis Platform. *Anal. Biochem*. **2020**, *610*, 113928. https://doi.org/10.1016/j.ab.2020.113928.

35. Charlermroj, R.; Phuengwas, S.; Makornwattana, M.; Sooksimuang, T.; Sahasithiwat, S.; Panchan, W.; Sukbangnop, W.; Elliott, C.T.; Karoonuthaisiri, N. Development of a Microarray Lateral Flow Strip Test Using a Luminescent Organic Compound for Multiplex Detection of Five Mycotoxins. *Talanta* **2021**, *233*, 122540. https://doi.org/10.1016/j.talanta.2021.122540.

36. Joshi, S.; Segarra-Fas, A.; Peters, J.; Zuilhof, H.; Beek, T.A. van Nielen, M.W.F. Multiplex Surface Plasmon Resonance Biosensing and Its Transferability towards Imaging Nanoplasmonics for Detection of Mycotoxins in Barley. *Analyst* **2016**, *141*, 1307–1318. https://doi.org/10.1039/c5an02512e.
37. Wei, T.; Ren, P.; Huang, L.; Ouyang, Z.; Wang, Z.; Kong, X.; Li, T.; Yin, Y.; Wu, Y.; He, Q. Simultaneous Detection of Aflatoxin B1, Ochratoxin A, Zearalenone and Deoxynivalenol in Corn and Wheat Using Surface Plasmon Resonance. *Food Chem*. **2019**, *300*, 125176. https://doi.org/10.1016/j.foodchem.2019.125176.
